# Gastric Ulcer with Penetration and Abscess in the Liver

**DOI:** 10.5334/jbsr.2683

**Published:** 2022-02-21

**Authors:** Pedro Marques

**Affiliations:** 1 Hospital Prof. Doutor Fernando Fonseca, PT

**Keywords:** peptic ulcer disease, fistulization/penetration, liver abscess, CT

## Abstract

**Teaching Point:** Aggressive peptic ulcer disease can be indistinguishable from malignancy on imaging, even presenting invasion of adjacent organs, which often implies histological characterization.

## Case History

**Figure 1:**
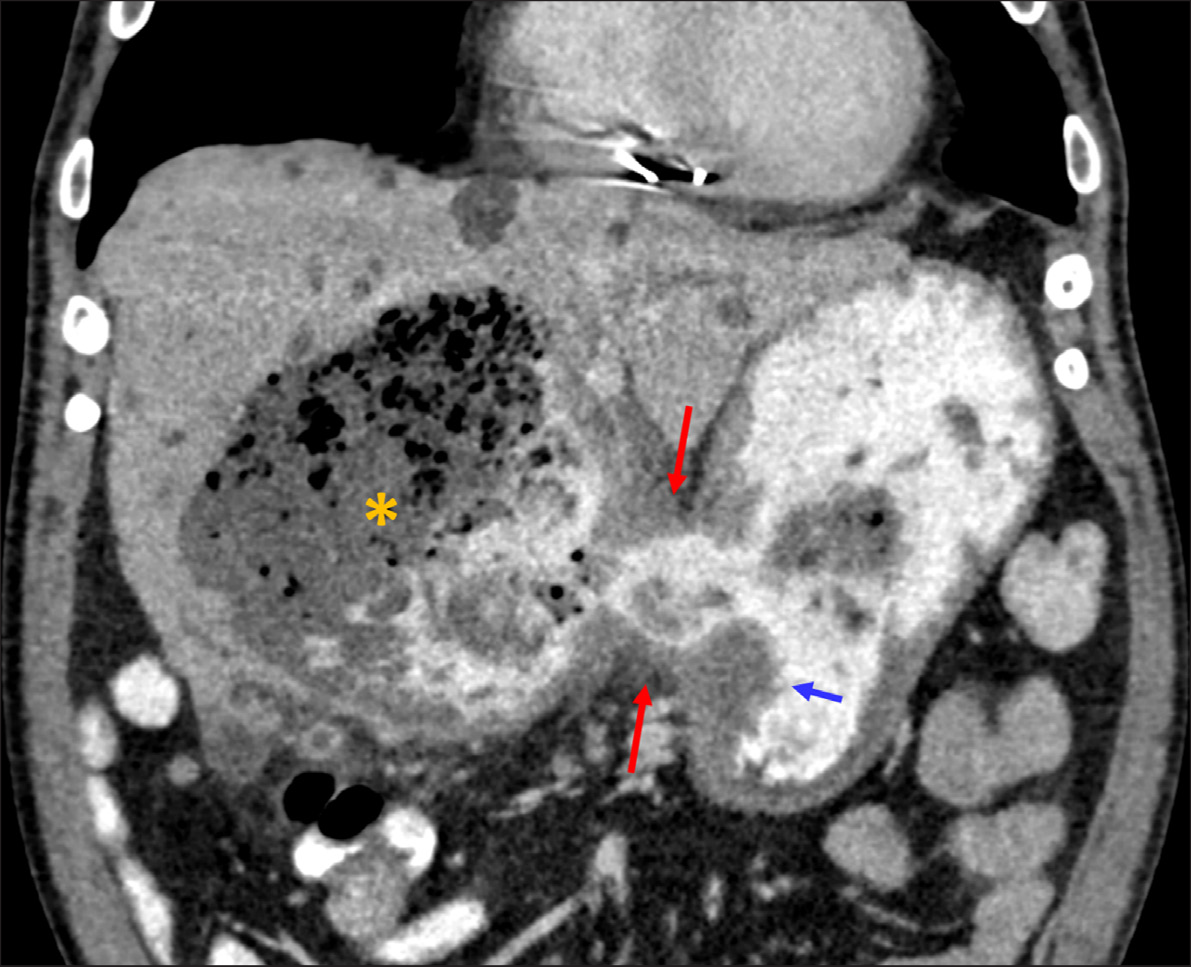


**Figure 2:**
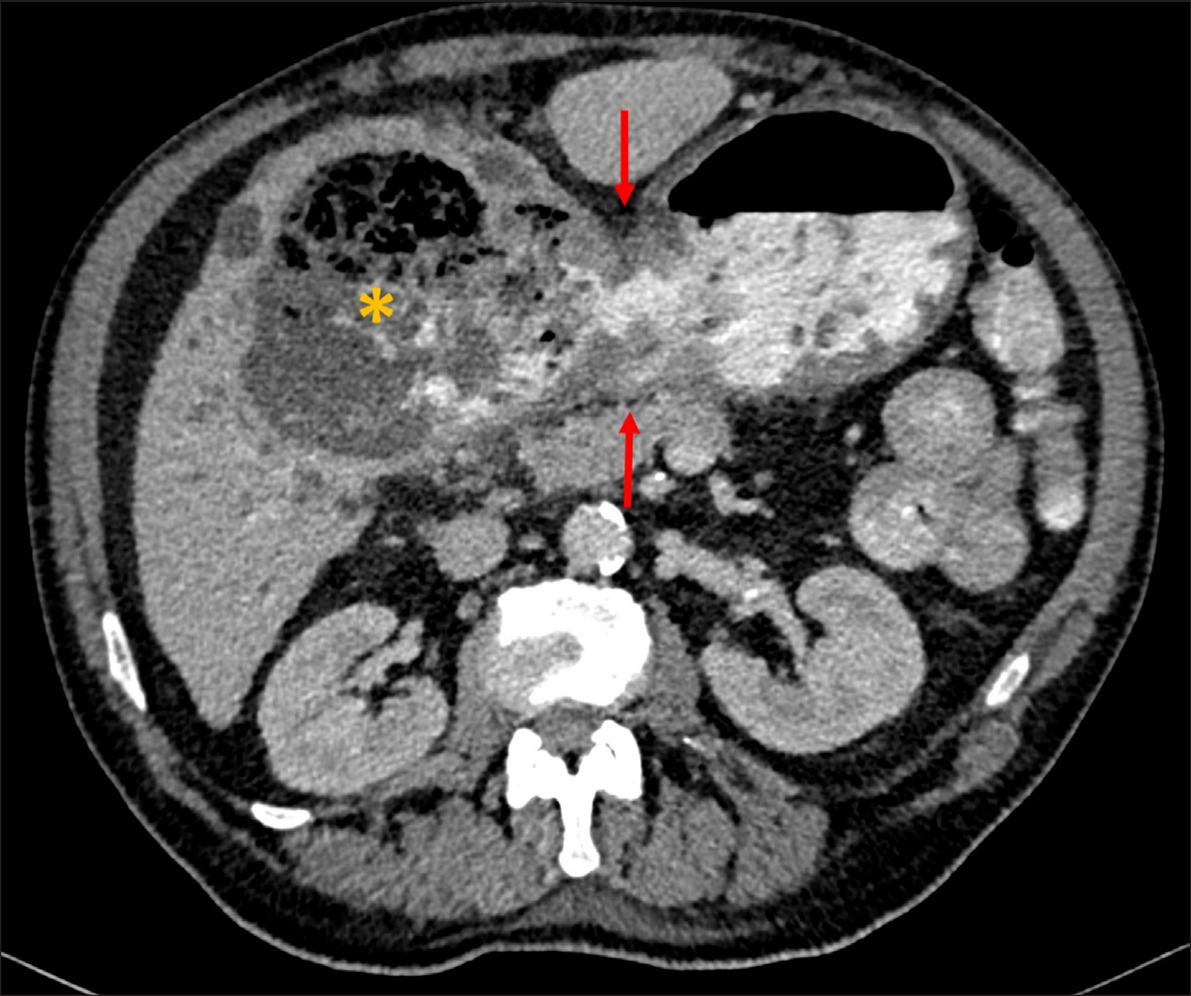


A 72-year-old male presented to the emergency department due to rectorrhagia on the night before. He reported dark stools over the previous month. His past medical history was relevant for peptic ulcer disease (PUD) and atrioventricular block. Physical examination revealed normal vital signs and the rectal examination showed neither bleeding, fissure, nor hemorrhoidal pathology. On blood analysis, severe anemia (5.8 g/dL), leukocytosis (12.4 × 10^9^/L), and increased C-reactive protein levels (16 mg/dL) were found. The patient underwent a blood transfusion and upper digestive endoscopy (UDE), which revealed a large ulcer next to the antrum, with pearly bottom and congestive edges. Biopsies were done. The patient underwent abdominopelvic computed tomography (CT) after intravenous and oral contrasts, which revealed an irregular thickening of the antral/inferior region of the lesser gastric curvature (blue arrow on coronal reformation, ***Figure 1***), with a 1.6 × 1.9 cm gastric wall defect communicating to the liver (red arrows on ***Figure 1*** and axial slice, ***Figure 2***). A large (>10 cm) hepatic collection was also identified, in continuity with the gastric lumen, predominantly containing liquid, multiple internal gas bubbles and oral contrast (asterisk on ***Figures 1–2***). These aspects were compatible with a large liver abscess due to penetration from a gastric ulcer. Absence of signs of peritoneal carcinomatosis and metastases favored benign etiology. The patient underwent subtotal gastrectomy with Billroth II reconstruction and liver abscess drainage. Pathological findings were of a benign peptic ulcer, with chronic atrophic antral gastritis and intestinal metaplasia, without evidence of dysplasia.

## Comment

PUD remains a highly prevalent disease, affecting 10% of the world population. Its main causes are intake of nonsteroidal anti-inflammatory drugs and *Helicobacter pylori* chronic infection. Diagnosis is currently established by UDE and histological correlation. Patients suffering from PUD generally present with mild to moderate chronic symptoms, sporadically aggravated by exacerbation of the disease. In these episodes, patients are often submitted to CT. Although mild PUD may not have imaging translation on CT, gastroduodenal inflammation may manifest as stratified wall thickening and stranding of the surrounding fat and adjacent lymph node enlargement (indirect signs) or mucosal enhancement discontinuity and luminal sacculation (direct signs) as in the present case. Acute complications of PUD such as hemorrhage and pneumoperitoneum (perforation) may equally be diagnosed on CT. Perforation may also lead to collection or fistulization/penetration to adjacent organs, which requires prompt diagnosis. PUD also increases the risk of gastric malignancies. Thus, although CT is not the first-line diagnostic technique, it has an essential role in the initial assessment and correct diagnosis, of both uncomplicated PUD and its complications [1].
